# Can Orthopedic Oncologists Predict Functional Outcome in Patients with Sarcoma after Limb Salvage Surgery in the Lower Limb? A Nationwide Study

**DOI:** 10.1155/2014/436598

**Published:** 2014-11-18

**Authors:** Sjoerd Kolk, Kevin Cox, Vivian Weerdesteyn, Gerjon Hannink, Jos Bramer, Sander Dijkstra, Paul Jutte, Joris Ploegmakers, Michiel van de Sande, Hendrik Schreuder, Nico Verdonschot, Ingrid van der Geest

**Affiliations:** ^1^Department of Rehabilitation, Radboud Institute for Health Sciences, Radboud University Medical Center, P.O. Box 9101, 6500 HB Nijmegen, The Netherlands; ^2^Orthopaedic Research Laboratory, Radboud Institute for Health Sciences, Radboud University Medical Center, P.O. Box 9101, 6500 HB Nijmegen, The Netherlands; ^3^Sint Maartenskliniek Research, P.O. Box 9011, 6500 GM Nijmegen, The Netherlands; ^4^Department of Orthopaedic Surgery, Amsterdam University Medical Centre, P.O. Box 22660, 1100 DD Amsterdam, The Netherlands; ^5^Department of Orthopaedic Surgery, Leiden University Medical Centre, P.O. Box 9600, 2300 RC Leiden, The Netherlands; ^6^Department of Orthopaedic Surgery, Groningen University Medical Centre, P.O. Box 30001, 9700 RB Groningen, The Netherlands; ^7^Department of Orthopaedic Surgery, Radboud Institute for Health Sciences, Radboud University Medical Center, P.O. Box 9101, 6500 HB Nijmegen, The Netherlands; ^8^Laboratory for Biomechanical Engineering, Faculty of Engineering Technology, University of Twente, P.O. Box 217, 7500 AE Enschede, The Netherlands

## Abstract

Accurate predictions of functional outcome after limb salvage surgery (LSS) in the lower limb are important for several reasons, including informing the patient preoperatively and, in some cases, deciding between amputation and LSS. This study aimed to elucidate the correlation between surgeon-predicted and patient-reported functional outcome of LSS in the Netherlands. Twenty-three patients (between six months and ten years after surgery) and five independent orthopedic oncologists completed the Toronto Extremity Salvage Score (TESS) and the RAND-36 physical functioning subscale (RAND-36 PFS). The orthopedic oncologists made their predictions based on case descriptions (including MRI scans) that reflected the preoperative status. The correlation between patient-reported and surgeon-predicted functional outcome was “very poor” to “poor” on both scores (*r* ^2^ values ranged from 0.014 to 0.354). Patient-reported functional outcome was generally underestimated, by 8.7% on the TESS and 8.3% on the RAND-36 PFS. The most difficult and least difficult tasks on the RAND-36 PFS were also the most difficult and least difficult to predict, respectively. Most questions had a “poor” intersurgeon agreement. It was difficult to accurately predict the patient-reported functional outcome of LSS. Surgeons' ability to predict functional scores can be improved the most by focusing on accurately predicting more demanding tasks.

## 1. Introduction

Limb salvage surgery (LSS) rather than amputation is the operation of choice in 70–85% of all malignant bone and soft tissue lower limb sarcomas [1, 2]. Since the oncological results for amputation and LSS in the surgical treatment of sarcomas are comparable [3, 4], the decision to perform an amputation or LSS is based on the tumor size, the tumor location, patient preferences, the expected risk of complications and multiple reoperations, and the expected functional outcome [3]. If it is surgically possible, LSS is generally the preferred treatment, unless a poor functional outcome is expected. It has been shown that the functional outcome of LSS is superior to amputation, with the exception of below-knee amputation, which yields a similar function as limb salvage [5]. The expected functional outcome of patients after LSS is thus an important part of the preoperative decision making process for the surgical treatment.

Several known predictors of functional outcome include tumor size, location, grade, bone resection, muscle involvement, use of radiotherapy, and motor nerve sacrifice [6]. The functional outcome is predicted by the surgeon based on these parameters combined with his/her clinical experience. However, to the best of our knowledge, there are no reports about how well surgeons are able to actually predict functional outcome after LSS. Insight into the level of accuracy of these predictions is important for several reasons. First, accurate predictions of functional outcome are highly relevant in informing the patient preoperatively about the expected final functional outcome. Second, in some cases, the predictions are helpful in deciding between amputation or LSS. Third, information about the correlation between predicted functional outcome and patient-reported functional outcome provides valuable information for surgeons in training.

In this study, (1) we aimed to establish whether orthopedic oncologists can accurately predict patient-reported functional outcome of LSS in the treatment of sarcoma in the lower limb in a selected group of patients. (2) We also examined whether there was a tendency to over- or underestimate patient-reported functional outcome. Additionally, (3) we sought to identify which items on the functional outcome scores were least difficult and which were most difficult to predict, and whether the surgeons agreed amongst themselves (interrater reliability) in their predictions.

## 2. Materials and Methods

### 2.1. Patients

**Figure 1:**
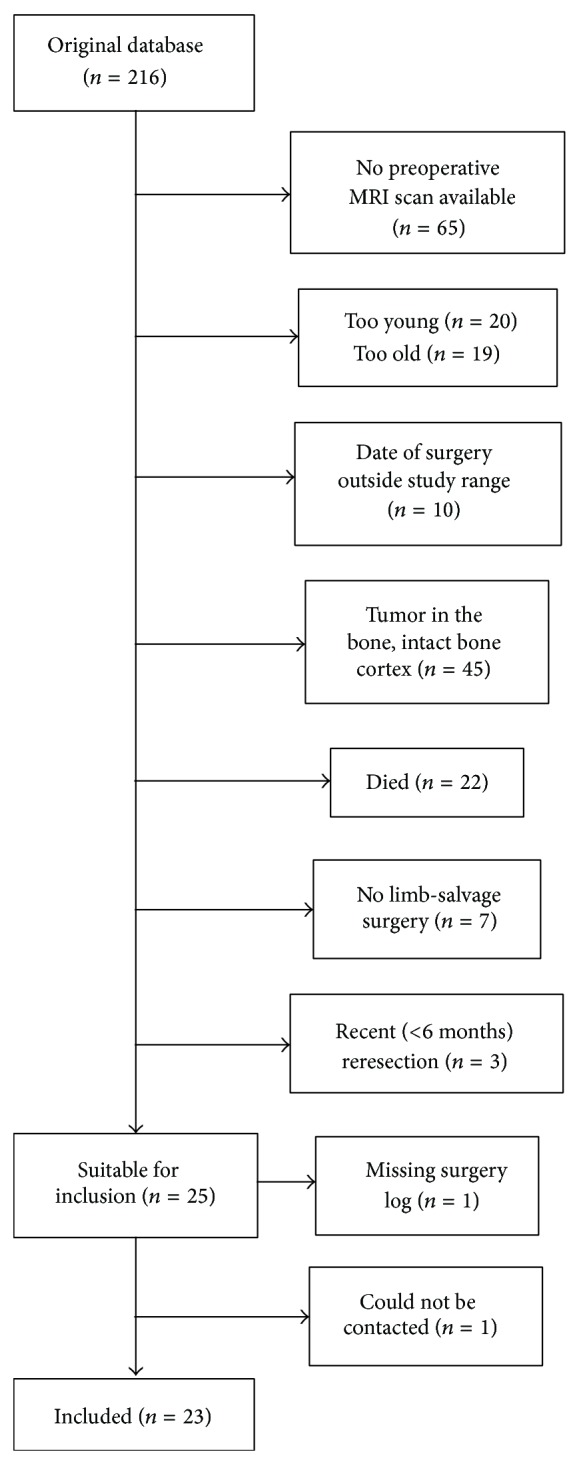
Flow chart of patient selection. Some patients fitted into multiple exclusion criteria (e.g., “no preoperative MRI scan available” and “too old”); in such cases, the patient was counted as belonging to the first of those exclusion criteria.

We selected patients who had undergone a LSS for a sarcoma in the lower limb from a database of orthopedic oncologic patients at the Department of Orthopedic Surgery of the Radboud University Medical Center (RUMC), Nijmegen, The Netherlands. The database contained 216 patients who had undergone LSS or amputation for any type of tumor in the hip or knee region. We selected patients using the following inclusion criteria: follow-up at least six months after the surgery (before July 1, 2012) for patients without adjuvant treatment and at least twelve months for patients with adjuvant treatment, a maximum follow-up of ten years (after February 1, 2003), and age between 18–70 years, and preoperative MRI scans had to be available. The follow-up of at least six months was chosen because functional scores tend to plateau within that time frame [6, 7]. We excluded patients who had a bone tumor with an intact cortical bone, as almost no functional deficits were expected to occur in those patients. Patients who had suffered local recurrence or complications that required reoperation in the last six months before the study were excluded. A flow chart of the patient selection is shown in Figure 1. Twenty-four patients were eligible for inclusion in the study, of whom 23 were successfully contacted. All 23 patients were included in the study. The study procedures were approved by the Local Ethical Committee of the RUMC. Written informed consent was obtained from all participants.

### 2.2. Materials

To evaluate the functional outcome, we used the Toronto Extremity Salvage Score (TESS) for the lower extremity and the RAND-36 physical functioning subscale (RAND-36 PFS). The TESS is a patient-reported questionnaire that has been specifically designed to measure the physical functional status of patients after limb-salvage surgery [7]. It contains 30 questions, and the final score ranges from 0% to 100%, 100% being the highest achievable score. The RAND-36 PFS is intended to measure physical functioning in any patient cohort [8, 9], which makes it more general than the TESS. Like the TESS, the RAND-36 PFS also is a patient-reported questionnaire. The RAND-36 PFS consists of ten questions, and the final score ranges from 0% to 100%, 100% being the highest achievable score. The RAND-36 PFS is identical to the SF-36 PFS. In addition to the TESS and RAND-36 PFS, we also used the RAND-36 pain subscale to examine postoperative pain levels. The RAND-36 pain subscale contains two questions; one regarding the amount of pain and one regarding the hindrance experienced due to pain when performing everyday activities in the previous four weeks [8, 9]. The final score ranges from 0% to 100%, where 100% represents no pain. We did not employ the Musculoskeletal Tumor Society score [10], as that score is not patient-reported and includes the domains of pain and emotional acceptance, which would have been impossible to predict solely on the basis of case descriptions.

**Figure 2:**
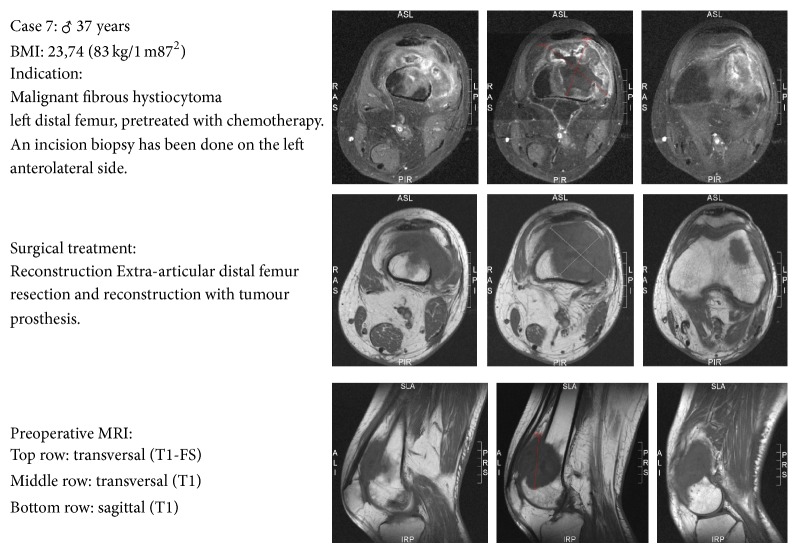
Example case as given to the orthopedic oncologists. This is patient 7 in Table 1.

A case description of each patient was made, which reflected the preoperative status of the patient. It contained the patient's age, sex, body mass index (BMI), tumor diagnosis, diagnostic MRI scans, a description of the performed surgical procedure for tumor resection and reconstruction, whether the patient had received adjuvant pre- or postoperative chemo- or radiotherapy, and whether there were any complications from the surgery (a case example is shown in Figure 2). The information did not include follow-up time. If a reresection had been performed, the preoperative MRI scans from before the primary resection surgery were provided, rather than those made after the local recurrence. The case descriptions were distributed through a central electronic platform. Whenever bone was removed, it was replaced by tumor prosthesis and/or an allograft.

### 2.3. Study Procedures

The patients were interviewed about their current functional status in a structured telephone call (done by KC, an independent researcher who was not a medical doctor), consisting of the TESS, the RAND-36 PFS, and the RAND-36 pain subscale. Five independent orthopedic oncologists (JB, PD, PJ, JP, and MvdS), working in one of the other three Dutch orthopedic oncologic referral centers (other than the RUMC) participated in the study. They were asked to give a prediction of the total TESS score (one percentage for the total functional status of the patient without addressing all separate items) and a prediction of the ten individual items of the RAND-36 PFS, based on the case descriptions. They had never been involved in the treatment of the patients and were unaware of their patient-reported functional outcome. All orthopedic oncologists were experienced and specialized in orthopedic oncology. They were familiar with the employed functional scales and were provided with a copy of the TESS questionnaire for reference.

### 2.4. Outcome Measures and Statistical Analyses

Descriptive statistics were calculated and stated as mean ± standard deviation. We compared the patient-reported and surgeon-predicted TESS and RAND-36 PFS scores in three ways.

First, Pearson correlations were calculated between the patients' reported scores and individual surgeon predicted scores, as well as for the average scores of all the surgeons combined. The squared correlation coefficient, *r* ^2^, (coefficient of determination), represents the variation in the values of the patient-reported outcome that can be explained by variations in the value of the surgeon-predicted outcome [11]. An *r* ^2^-value of 0.75–1.00 was interpreted as a “very good” prediction, 0.50–0.74 as “good,” 0.25–0.49 as “poor,” and 0-0.24 as “very poor.” The *r* ^2^-values were considered the primary outcome measure.

Second, the mean differences and 95% confidence intervals (95% CI) between the patient-reported scores on the TESS and RAND-36 PFS and the surgeon-predicted scores were calculated to reveal whether the predictions had a bias towards being too optimistic or pessimistic.

Third, the agreement between patient-reported and the median surgeon-predicted answers to the separate questions of the RAND-36 PFS were examined using percent agreement and Gwet's agreement coefficient (AC1). Compared with Cohen's Kappa [12, 13], Gwet's AC1 has a more stable interrater reliability and is less affected by prevalence and marginal probability [14]. This allowed us to identify which questions were the least difficult and most difficult to predict. The intersurgeon agreement on each separate question was also calculated, using percent agreement and Gwet's AC1. To calculate the intersurgeon agreement on the TESS, we used the intraclass correlation coefficient (ICC; absolute single measure/absolute agreement). Agreement coefficients below 0.40 were considered to represent a “poor” agreement; between 0.40 and 0.59 “fair”; between 0.60 and 0.74 “good”; and between 0.75 and 1.00 “excellent,” analogous to commonly used guidelines for interexaminer agreement [15].

The associations between each separate variable (age, sex, BMI, pain, and time since surgery) and patient-reported TESS and RAND-36 were examined using univariate regression analyses to examine whether they were associated with the functional outcome scores.

Matlab R2011a (The Mathworks, Natick, MA, USA) and R version 3.0.2. [16] were used for the statistical analyses.

## 3. Results

### 3.1. Patients

The characteristics of all 23 patients are listed in Table 1. The age at the time of surgery was 39.9 ± 18.8 years and the time after surgery was 47 ± 27 months. All patients were ambulatory and able to at least walk short distances without a walking aid. Two patients (cases 10 and 21) had undergone a reresection; this was mentioned in the case file. All other patients had not suffered from local recurrence or complications that required follow-up surgery. The mean patient-reported scores were TESS 87.0 ± 12.1, RAND-36 PFS 73.3 ± 18.7, and RAND-36 pain subscale 85.5 ± 24.7.

### 3.2. Surgeon Predictions—TESS

**Figure 3:**
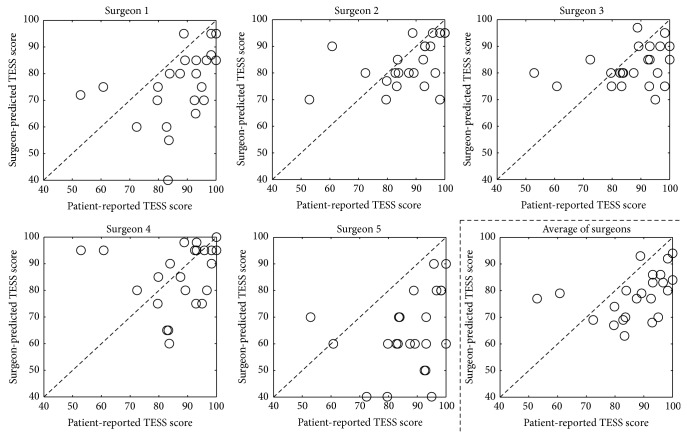
Scatter plots of patient-reported outcome and orthopedic tumor surgeon predictions on the Toronto Extremity Salvage Scale (TESS). The dashed lines indicate a hypothetical perfect correlation; if a patient case lies above or below this line, the functional outcome was overestimated or underestimated, respectively.

The surgeon-predicted scores and their correlations with the patient-reported scores of all five surgeons and the average predictions of all surgeons on the TESS are shown in Figure 3 and in Table 2. The correlations with the patient-reported scores were “very poor” for all surgeons, with the best correlation for surgeon 2 (*r* ^2^ = 0.185). The TESS was underestimated for most patient cases (Figure 3); the mean underestimation ranged from 1.5 to 22.6 percentage points (Table 2). The correlations with the patient-reported TESS formed by averaging all five surgeons' predictions were “very poor” (*r* ^2^ = 0.159) and underestimated patient-reported functional outcome by 8.7 (95% CI: 3.62–13.7) percentage points. The intersurgeon agreement on the TESS was “poor” with an ICC of 0.29 (95% CI: 0.10–0.53).

### 3.3. Surgeon Predictions—RAND-36 PFS

**Figure 4:**
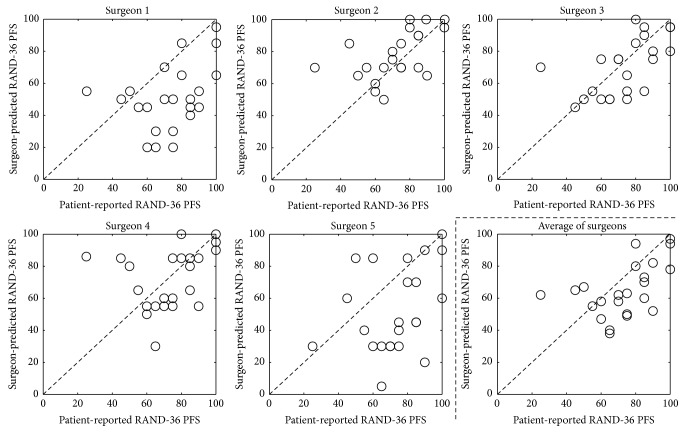
Scatter plots of patient-reported outcome and orthopedic tumor surgeon predictions on the RAND-36 physical functioning subscale (RAND-36 PFS). The dashed lines indicate a hypothetical perfect correlation; if a patient case lies above or below this line, the functional outcome was overestimated or underestimated, respectively.

The surgeon-predicted RAND-36 PFS scores and their correlations with the patient-reported scores are shown in Figure 4 and in Table 2. The correlations to the patient-reported scores were either “very poor” (surgeons 1, 4, and 5) or “poor” (surgeons 2 and 3). Surgeon 3's predictions had the highest correlation with the patient-reported scores (*r* ^2^ = 0.354). The patient-reported RAND-36 PFS score was underestimated by all surgeons, except for surgeon 2 (5.4 percentage points overestimation) (Table 2). The average correlations with the patient-reported scores were “poor” (*r* ^2^ = 0.255) and underestimated patient-reported functional outcome by 8.3 (95% CI: 0.64–16.0) percentage points.

In the analysis of the individual questions that make up the RAND-36 PFS, “*Climbing several flights of stairs*” and “*Walking more than a mile*” were the most difficult items to predict, with “poor” agreement coefficients (AC1) of 0.15 and 0.19, respectively, between surgeon-predicted and patient-reported scores (Table 3). “*Walking one block*” and “*Bathing or dressing yourself*” were the least difficult items to predict, with “excellent” agreement coefficients of 0.81 and 0.76, respectively, between surgeon-predicted and patient-reported scores. Similar to the overall RAND-36 PFS scores, most of its separate questions were underestimated; only two questions were overestimated (“*Bending, kneeling, or stooping*” and “*Lifting or carrying groceries*”).

On most questions of the RAND-36 PFS, the intersurgeon agreement coefficient was “poor,” but there was a “fair” agreement on “*Bathing or dressing yourself*” and “*Moderate activities*” and a “good” agreement on “*Vigorous activities*” and “*Walking one block*” (Table 3).

### 3.4. Other Potential Predictors

No correlations were found between the TESS or RAND-36 PFS and any of the potential predicting factors (Table 4).

## 4. Discussion

This national survey aimed to investigate how well orthopedic oncologists are able to predict the patient-reported functional outcome of patients that had undergone LSS in the lower limb. We found “very poor” to “poor” correlations between patient-reported outcomes and surgeon-predicted outcomes on both the TESS and the RAND-36 PFS. The orthopedic oncologists tended to underestimate patient-reported functional outcome on both scales. The most difficult tasks on the RAND-36 PFS were also the most difficult to predict, whereas, for the least difficult tasks, it was easy to predict that these could be performed without substantial limitations by nearly all patients. The intersurgeon agreement on the RAND-36 PFS questions was mostly “poor” but was “good” for some of the most and least demanding tasks. None of the potentially predicting factors were related to the primary outcome measures.

Our results indicate that it was difficult for the participating orthopedic oncologists to accurately predict the patient-reported functional outcome of limb salvage surgery. On the TESS, for instance, the coefficients of determination (*r* ^2^) between patient-reported and surgeon-predicted outcomes were lower than 0.20, indicating that less than 20% of the variance in TESS could be explained by the predictions made by the orthopedic oncologists. We did not expect such a poor predictive ability, considering the experience level of the orthopedic oncologists with limb salvage surgery. Several aspects may underlie this seemingly rather poor predictive ability.

First, each limb salvage patient presents a unique case in terms of anatomical involvement. Even in patients with the same type of tumor at a similar location, for instance, the distal femur, final functional results can differ to a large extent. In part, this depends on the amount and precise location of soft tissue involvement, which may have been difficult to see from the limited set of MRI images in the case files. Moreover, patients are unique in terms of adaptive capacity. The adaptation of the patient to the new anatomical and sensorimotor situation plays a large role in the recovery of function [17]. The amount of adaptive capacity may have been hard or impossible to estimate by the orthopedic oncologists from the case files. Second, we measured functional outcome with questionnaires, which are inherently subjective. Thus, the patients' own perception of functioning may have played a large role in the functional outcome score. It might be that functional outcome measured by objective means, such as, for example, gait analysis, more closely reflects the orthopedic oncologists' predictions. Third, in the case files, we mimicked as well as possible the information typically available preoperatively to the surgeon in a clinical setting, but the study design did not permit the independent surgeons to review the medical history of the patients nor perform a physical examination before the surgery. As such, predictions of patient-reported functional outcome in a “real” clinical setting (e.g., including a physical examination) might be more accurate than those made in this study. Fourth, patients who had a bone tumor with an intact cortical bone were not included; the patient-reported functional outcome in those patients would potentially have been less difficult to predict than that in the patients with larger tumors.

The poor predictive ability raises the question of which other factors determine functional outcome in limb saving surgery and to what degree. Davis et al. showed that large tumor size, deep lesions, high grade tumor, use of radiotherapy, bone resection, and motor nerve sacrifice are significantly related to increased disability on the TESS [6]. In their study, those combined parameters were able to predict 20% of the variance in TESS score. This is in the same order of magnitude as the presently reported results, indicating that the surgeons were unable to “add” predictive value on top of the given parameters in the case files. The rehabilitation protocol may also have an effect on functional outcome; Shehadeh et al. showed that adherence to a strict rehabilitation protocol after limb salvage surgery led to a relatively high level of functional outcome compared with other studies [18]. If we interpret our findings concurrent with those of Davis et al. and Shehadeh et al., it appears that still a large percentage of functional outcome cannot be predicted by the surgeon nor by anatomical and surgery or adjuvant therapy-related factors nor by rehabilitation protocols. Other factors that may play a significant role in the patient-reported functional outcome include the preoperative physical and mental state of the patient. For example, a patient who is highly motivated and athletic may recover to a far higher level of functioning than one who is less motivated and leads a sedentary lifestyle. From this perspective, one may intuitively expect a correlation between patient-reported functional outcome and age or BMI, but we did not find this (Table 4). Further studies are required to clarify the role each factor plays in patient-reported functional outcome after limb salvage surgery.

The orthopedic oncologists tended to underestimate patient-reported functional outcome on both the TESS and the RAND-36 PFS. Thus, it appears that the patients adapted to the new anatomical and functional situation better than the surgeons predicted. It is possible that this is due to some surgeons being used to picturing a somewhat more pessimistic scenario to their patients so that the actual achieved functional result exceeds the patients' expectations. However, we specifically instructed the surgeons to provide their most accurate predictions of patient-reported functional outcome, rather than to provide predictions that they would share with patients. As for clinical relevance, we did not set a specific threshold, but the underestimation of patient-reported functional outcome on both the TESS and the RAND-36 PFS was rather consistent, as demonstrated by the 95% confidence intervals that did not pass through zero.

Interestingly, we found that the “*Walking one block*” question was the least difficult to predict, whereas the “*Walking more than a mile*” question was one of the most difficult questions to predict (only “*Climbing several flights of stairs*” was more difficult to predict). It appears that the ultimate level of function that is reached in patients is hard to predict, whereas it is easier to predict lower levels of function. Thus, surgeons' ability to predict functional scores can be improved the most by focusing on accurately predicting more demanding tasks. Additional improvement might be gained by analyzing the prediction for the “*Bending, kneeling, or stooping*” question. If the prediction for this question did not match with the patient-reported outcome, it was mostly overestimated (43.5% of cases). This overestimation breaks with the general trend to underestimate patient-reported functional outcome and indicates that bending the knees is more difficult to do for patients than the median surgeon predicted.

The intersurgeon agreement on most RAND-36 PFS questions was “poor,” indicating that there was a high intersurgeon variability in the predictions to the questions. Notable exceptions were “*Walking one block*” and “*Vigorous activities*,” with “good” intersurgeon agreement. The prior arguably is the least difficult activity on the scale, whereas the latter represents the most demanding activities on the scale (including running, heavy lifting, and strenuous sports). However, this does not imply that there was also a high agreement with the patient-reported outcome; “*Vigorous activities*” had only a “fair” agreement with the patient-reported score. “*Walking one block*,” on the other hand, was the only question that had both an “excellent” agreement with the patient-reported score as well as a “good” intersurgeon agreement. This might be due to the surgeons' familiarity with predicting this basic level of functional outcome or because being able to walk at least short distances is considered one of the criteria for attempting limb salvage surgery, and most patients indeed achieved that goal.

This study has some limitations. First, the surgeons only predicted the total TESS score, instead of predicting each of the 30 questions that comprise the score. This was done because some questions were already present in the much shorter RAND-36 PFS, and to reduce the time it would take the surgeons to predict the 23 cases. Second, we used a translated version of the TESS which has not been validated in Dutch. However, as the TESS is the gold standard assessment tool after limb salvage surgery, we decided to use it [19]. The RAND-36 PFS has been validated in Dutch [8, 9], and its results showed the same trend in the comparisons as the translated TESS. Third, we found a wide range of patient-reported functional outcome scores, including in patients that had undergone similar surgery. Of course, each case is unique, but the perception of effort required to perform the activities in the questionnaires and the interpretation of the questions can vary between patients. Measuring actual functional outcome (e.g., in a movement laboratory or by observing patients in their home setting) could yield more knowledge of actual functioning, eliminate the subjectivity inherent in questionnaires, and establish the construct validity of the employed functional scoring systems. Fourth, the surgeons predicted the functional outcome based on a case description without being allowed to review the medical history of the patients or perform a physical examination. The time since surgery was also not provided, which might have negatively affected the predictions. This, however, does not explain the large differences found between predicted and patient-reported functional outcome nor does it explain the differences in predictions between surgeons. Furthermore, there was no correlation between the patient-reported functional scores and the time since surgery (Table 4).

## 5. Conclusions

It was difficult for the participating orthopedic oncologists to accurately predict the patient-reported functional outcome of limb salvage surgery. Patient-reported functional outcome tended to recover to a higher level than the surgeons predicted. The ultimate level of function that the patients reached was hard to predict, whereas it was easier to predict lower levels of function. Thus, surgeons' ability to predict functional scores can be improved the most by focusing on accurately predicting more demanding tasks. Intersurgeon agreement to most questions was “poor,” indicating the high variability in the surgeons' predictions, and, possibly, treatment decisions. The poor predicting ability warrants research into objective tools to assist orthopedic oncologists in the decision making process. Such tools could include, for instance, computational musculoskeletal models that prospectively calculate whether enough muscle strength remains to perform activities of daily living.

## Figures and Tables

**Table 1:** Patient characteristics, indication, tumor location, surgical treatment, adjuvant therapy, and functional scores.

Pat. number	Gender, age (y)	BMI (kg/m^2^)	Indication	Tumor location	Side	Surgical treatment	Adjuvant therapy	Time post-OR (months)	TESS score	RAND-36 phys. func.	RAND-36 pain
1	M, 50	26.0	Paget's osteosarcoma	Distal femur	Left	Extra-articular distal femur resection including part of the quadriceps muscle. Reconstruction with tumor prosthesis and biceps femoris tendon transposition for quadriceps reconstruction	Pre- and postoperative chemotherapy	67	79.6	65.0	89.8

2	M, 36	21.4	Clear cell chondrosarcoma	Proximal femur	Right	Proximal femur resection, including the greater trochanter Reconstruction with tumor prosthesis		28	89.2	70.0	67.3

3	V, 44	28.3	Myxoid liposarcoma next to the medial femoral epicondyle	Distal femur	Left	Soft tissue tumor resection, including the medial collateral ligament Reconstruction of the pes anserinus		75	98.3	100.0	100.0

4	V, 63	25.2	Sarcoma not otherwise specified tibial tuberosity	Proximal tibia	Left	Proximal tibia resection Reconstruction with allograft and tumor prosthesis. Soft tissue closure with gastrocnemius transfer and split skin graft		62	95	75.0	100.0

5	V, 60	25.4	Liposarcoma	Medial thigh	Left	Resection soft-tissue tumor		21	88.8	80.0	100.0

6	V, 66	25.2	Parosteal osteosarcoma	Distal femur	Right	Distal femur resection Reconstruction with tumor prosthesis		12	98.3	70.0	100.0

7	M, 37	23.7	Sarcoma not otherwise specified	Distal femur	Left	Extra-articular distal femur resection and reconstruction with tumor prosthesis	Pre- and postoperative chemotherapy	65	92.9	90.0	100.0

8	V, 60	24.1	Soft tissue sarcoma adductor muscles compartment	Medial thigh	Right	En block resection	Preoperative chemotherapy	14	93.1	90.0	100.0

9	V, 41	34.3	Chondrosarcoma grade 2	Proximal tibia	Left	Proximal tibia resection Reconstruction with tumor prosthesis		10	83.3	75.0	100.0

10	M, 47	28.7	Soft tissue sarcoma; previous incomplete resection	Thigh	Right	Reresection		105	96.7	85.0	79.6

11	M, 15	19.2	Osseous lipoma like liposarcoma	Proximal femur	Right	Proximal femur resection (osteotomy at 250 mm) and reconstruction with tumor prosthesis	Preoperative chemo- and radiotherapy	46	82.8	60.0	79.6

12	V, 16	24.5	Osteosarcoma	Tibia shaft	Right	Segment resection tibia shaft, saving tibia epiphysis Reconstruction with allograft, intramedullary pen, cement, and plate osteosynthesis	Pre- and postoperative chemotherapy	30	92.5	75.0	100.0

13	V, 15	26.9	Osteosarcoma	Distal femur	Right	Distal femur resection (235 mm), reconstruction with tumor prosthesis	Pre- and postoperative chemotherapy	79	72.4	25.0	100.0

14	V, 65	25.9	Osteosarcoma	Distal femur	Left	Distal femur resection, reconstruction with tumor prosthesis		55	79.8	60.0	0.0

15	V, 39	24.9	Synovial sarcoma between proximal fibula and tibia	Proximal fibula	Left	Proximal fibula resection, including lateral cortex of tibia Reconstruction with plate and cement.		40	93.1	80.0	100.0

16	M, 59	25.8	Chondrosarcoma grade 2	Distal femur	Left	Distal femur resection, reconstruction with tumor prosthesis		7	52.9	50.0	79.6

17	V, 59	23.8	Soft tissue sarcoma not otherwise specified	Dorsal thigh	Right	Resection soft tissue tumor	Postoperative radiotherapy	51	60.8	45.0	100.0

18	M, 15	26.6	Telangiectatic osteosarcoma	Distal femur	Left	Distal femur resection, reconstruction with tumor prosthesis	Pre- and postoperative chemotherapy	84	83.6	65.0	57.1

19	M, 21	24.2	Osteosarcoma	Distal femur	Left	Distal femur resection, reconstruction with tumor prosthesis	Pre- and postoperative chemotherapy	12	83.9	55.0	44.9

20	V, 30	21.7	Myxoid liposarcoma	Rectus femoris	Left	Resection soft tissue sarcoma		45	100.0	100.0	100.0

21	V, 49	26.8	Liposarcoma, previous incomplete resection	Anterolateral thigh	Right	Reresection (last part vastus lateralis anteromedial and distal)	Postoperative radiotherapy	50	95.8	90.0	69.4

22	M, 17	22.6	Osteosarcoma	Lateral femoral epicondyles	Right	Distal femur resection, reconstruction with tumor prosthesis	Pre- and postoperative chemotherapy	50	87.5	85.0	100.0

23	M, 14	21.5	Ewing's sarcoma	Diaphysis femur	Right	Segment resection right femur Reconstruction with allograft, intramedullary nail, and plate osteosynthesis	Pre- and postoperative chemotherapy	77	100.0	100.0	100.0

**Table 2:** Patient-reported and surgeon-predicted mean TESS and RAND-36 PFS scores and coefficients of determination for TESS and RAND-36 PFS scores.

	TESS		RAND-36 PFS	
	Mean score^a^	*r* ^2^	Mean score^a^	*r* ^2^
Patient-reported	87.0 (12.1)		73.3 (18.7)	
Surgeon 1	75.6 (13.6)	0.167	50.9 (20.3)	0.142
Surgeon 2	83.6 (8.6)	0.185	78.7 (15.5)	0.336
Surgeon 3	82.5 (7.1)	0.096	70.2 (17.9)	0.354
Surgeon 4	85.5 (11.9)	0.014	72.2 (18.7)	0.081
Surgeon 5	64.3 (14.7)	0.088	52.8 (26.8)	0.118

Average of surgeons	78.3 (8.7)	0.159	65.0 (16.6)	0.255

^a^Scores are reported as mean (SD).

**Table 3:** Analysis of separate questions on the RAND-36 physical functioning subscale.

	Patient-reported score			Surgeon-predicted score			Patients versus median surgeon				Intersurgeon agreement	
	“Yes, limited a lot”	“Yes, limited a little”	“No, not limited at all”	“Yes, limited a lot”	“Yes, limited a little”	“No, not limited at all”	Percent agreement	Percent of cases underestimated	Percent of cases overestimated	AC1	Percent agreement	AC1
Vigorous activities	14	6	3	14	8	1	60.9%	26.1%	13.0%	0.47	70.0%	0.60
Moderate activities	0	7	16	0	16	7	60.9%	39.1%	0%	0.48	66.5%	0.55
Lifting or carrying groceries	2	7	14	0	9	14	65.2%	13.0%	21.7%	0.53	50.4%	0.32
Climbing several flights of stairs	2	10	11	4	16	3	39.1%	47.8%	13.0%	0.15	44.3%	0.20
Climbing one flight of stairs	1	4	18	0	9	14	52.2%	30.4%	17.4%	0.39	52.2%	0.35
Bending, kneeling, or stooping	6	13	4	0	16	7	52.2%	4.3%	43.5%	0.35	49.1%	0.28
Walking more than a mile	5	6	12	1	17	5	43.4%	34.8%	21.7%	0.19	54.3%	0.36
Walking several blocks	1	4	18	0	10	13	52.2%	34.8%	13.0%	0.38	50.9%	0.33
Walking one block	1	1	21	0	2	21	82.6%	8.7%	8.7%	0.81	67.8%	0.61
Bathing or dressing yourself	0	1	22	0	4	19	78.3%	17.4%	4.3%	0.76	60.0%	0.50

**Table 4:** Coefficients of determination between patient characteristics and functional outcomes (TESS and RAND-36 physical functioning subscale).

	TESS	RAND-36 PFS
	*r* ^2^	*r* ^2^
Age	0.011	0.001
Sex	0.024	0.001
Body mass index	0.008	0.003
Pain (RAND-36 pain subscale)	0.042	0.079
Time since surgery	0.036	0.015
